# Enduring effects of psychotherapy, antidepressants and their combination for depression: a systematic review and meta-analysis

**DOI:** 10.3389/fpsyt.2024.1415905

**Published:** 2024-11-27

**Authors:** Ulrich Voderholzer, Barbara B. Barton, Matthias Favreau, Eva M. Zisler, Winfried Rief, Marcel Wilhelm, Elisabeth Schramm

**Affiliations:** ^1^ Department of Psychiatry and Psychotherapy, University Hospital of Munich, Ludwig-Maximilians-University Munich (LMU) Munich, Munich, Germany; ^2^ Department of Psychiatry and Psychotherapy, University Hospital of Freiburg, Freilburg, Germany; ^3^ Schoen Clinic Roseneck, Prien am Chiemsee, Germany; ^4^ Department of Clinical Psychology and Psychotherapy, University of Marburg, Marburg, Germany; ^5^ University Psychiatric Clinics (UPK), Basel, Switzerland

**Keywords:** antidepressants, carry-over effect, depression, long-term, psychotherapy, follow-up

## Abstract

**Introduction:**

Although depressive disorders are frequently associated with relapses, the sustained efficacy of therapies after their termination has been insufficiently investigated.

**Objective:**

The aim of this study was to evaluate the current evidence of enduring effects of psychotherapy, antidepressants and their combination after the end of treatment.

**Methods:**

PubMed and PsychINFO were systematically screened according to PRISMA guidelines (except for preregistration). Only randomized controlled trials (RCTs) between 1980 and 2022 comparing the efficacy of psychotherapy, antidepressants and their combination in adult depression at follow-up at least 12 months after termination of therapy, which could be acute phase, maintenance or relapse prevention therapy, were included. Risk of bias was assessed by using the Cochrane risk of bias tool.

**Results:**

In total 19 RCTs with a total of 1154 participants were included. Psychotherapy was significantly superior to pharmacotherapy regarding relapse rates and Beck Depression Inventory scores at follow-up after acute treatment in two of nine RCTs. Combined treatment performed significantly better than pharmacotherapy, but not psychotherapy, regarding relapse and remission in five out of nine RCTs at least 12 months after treatment termination. Pairwise meta-analyses indicated a superiority of combined treatment compared to pharmacotherapy alone regarding relapse, recurrence, and rehospitalization rates (RR=0.60, 95%-CI: 0.37-0.97, p=.041) and for psychotherapy compared to pharmacotherapy alone regarding relapse and recurrence rates (RR=0.58, 95%-CI: 0.38-0.89, p=.023), however comparative treatment effects between psychotherapy and combined treatment were insignificant.

**Conclusions:**

Current findings suggest a superiority of psychotherapy and combined treatment over pharmacotherapy alone in major depressive disorder depression. Major limitations were a low number of studies reporting follow-up data after termination of study periods and a heterogeneity in definitions of treatment outcomes. Practice guidelines and participatory decision-making processes for the choice of treatment should consider the current knowledge on long-term effects of antidepressant therapy methods more than has been the case to date.

## Introduction

Major Depressive Disorder is associated with a high risk of relapse and recurrence of about 50% after the first depressive episode which increases with each subsequent episode and approximately 30% of depressed patients suffer from chronic depression (1–5). According to the DSM-5 (6) chronic depression or persistent depressive disorder (PDD) is defined by suffering from depressive symptoms for at least two years without a period of more than two months without those symptoms. Whether psychotherapy (PT) or pharmacotherapy or a combination of both is more effective in terms of sustainable improvement of depressive symptoms is most relevant to reduce the risk of experiencing relapse or recurrence. However, enduring treatment effects that persist beyond treatment termination have been insufficiently investigated for depressive disorders up to date. In other words, less is known about how efficient treatments are after having been off the treatment for a specific period of time, which is why these effects are also known as carry-over effects.

While the overall efficacy of PT and antidepressants (AD) as an acute treatment for depression is comparable, a combination of both is superior compared to either monotherapy (7–11). For PDD, meta-analyses found a combination of the Cognitive Behavioral Analysis System of Psychotherapy (CBASP) and pharmacotherapy to be superior to pharmacotherapy alone after acute treatment (12, 13).

As only a few studies have investigated the influence of different acute therapies on long-term outcomes in major depressive disorder, current guideline recommendations are mostly based on short-term efficacy of acute treatment. Greater knowledge about the long-term efficacy of depression treatments is highly needed both for the health care system as well as for clinicians and patients to make thorough informed decisions (14–17). In studies different outcome types are reported such as response, remission, recovery, relapse, or recurrence. While definitions in studies often vary, there are suggestions as how to define these outcomes (Paykel et al., 2008). According to Paykel et al. (2008) patients *respond* to treatment if they experience clinical improvements in their symptoms, while *remission* means that the immediate episode of the disease is over and the patient has low symptom levels or none at all. Further, *recovery* follows remission for a period of at least 4 months, *relapse* occurs when the depressive episode returns after remission, while *recurrence* defines the onset of a new depressive episode after a recovery (Paykel et al., 2008).”

Previous meta-analyses reported an advantage of combined psycho- and pharmacotherapeutic treatment over pharmacotherapy alone in the long-term (8, 18–20). In addition, some studies suggest that psychotherapy alone is superior to pharmacotherapy alone and equivalent to a combination of both on the long run (8, 18, 20). A current meta-analysis by Guidi and Fava (21) revealed a significantly reduced pooled risk ratio of relapse/recurrence at follow-up in major depressive disorder for the sequential combination of psychotherapy alone or of the combination of psychotherapy and pharmacotherapy following response to acute-phase pharmacotherapy. Furukawa, Shinohara (2) showed higher rates of sustained response for psychotherapy and combined treatment compared to pharmacotherapy independent of maintenance treatment. However, these meta-analyses either did not specifically examine long-term effects persisting after completion of treatment or focused on outcomes like response, remission or recovery only. Cuijpers, Noma (8) concluded in their meta-analysis that long-term effects of different treatments are still unknown as in most studies the studied pharmacotherapy or psychotherapy was continued during the follow-up or tapered out, while only in a few studies the setting was naturalistic, that is patients might have received further treatment but the study drug or study psychotherapy was not continued. “In addition, also outcomes like relapse or recurrence rates should be considered when evaluating long-term efficacy.

The aim of this study was to examine the current evidence for enduring treatment effects at least 12 months after treatment termination including all types of evidenced-based psychotherapy compared to pharmacotherapy alone and the combination of both. It was not the aim of our analyses to answer the questions which specific type of psychotherapy or which specific medication is superior.

## Methods

### Search strategy

According to the Preferred Reporting Items for Systematic Reviews and Meta-analyses (PRISMA) guidelines, we searched Pubmed and PsycINFO for RCTs published between Jan 1, 1980, and Nov 1, 2022, comparing the long-term efficacy of evidence-based psychotherapy, antidepressants, and their combination in the treatment of major depressive disorder and chronic depression. Search terms used for literature research are provided in the **appendix (A.1)**, but in brief the following terms were used (depressive [title] OR depression [title] OR depressed [title] OR “affective disorder” [title] OR “major depressive disorder” [title] OR “persistent depression” [title] OR “chronic depression” [title] OR “recurrent depression” [title] OR “persistent depressive disorder” [title] OR MDD [title]) AND (“long-term” OR “enduring effect” OR “lasting effect” OR “persist” OR relapse OR maintenance OR stability OR stable OR recurrence OR continuation) AND (“follow-up”) AND (CBT OR CT OR IPT OR MBCT OR ACT OR MBSR OR CBASP OR psychodynamic OR “wellbeing therapy” OR psychoanal* OR psychothera* OR “Cognitive behavioral analysis system of psychotherapy” OR “Cognitive behavioural analysis system of psychotherapy) AND (antidepressant OR pharma* OR SSRI OR SNRI OR NARI OR MAO OR TCA OR TeCA) AND (random* OR control). We report on results measured at a specific follow-up point and on measurements (e.g., relapse rates) during the follow-up phase separately.

### Eligibility *criteria*

Only studies that included patients with at least 18 years of age and a major depressive disorder as well as PDD or recurrent depression were selected. Studies in which depression was treated as a comorbidity to other somatic disorders e.g., diabetes or cancer, were excluded. Included were RCTs in which psychotherapy (individual or group therapy, no couple therapy), antidepressants or the combination of both were compared to each other in terms of enduring effects at least 12 months after treatment termination (**Table 1**). We differentiated between acute treatment, maintenance treatment and relapse prevention. Patients receiving acute treatment started a new treatment, patients receiving maintenance treatment had already received the studied treatment but continued the treatment usually in a lower frequency and patients receiving relapse prevention had already terminated their initial therapy but received treatment to prevent a relapse. No matter what kind of treatment, acute/maintenance/relapse prevention was studied, the treatment had to be terminated and a follow-up measure had to take place either during or after 12 months. We accepted only studies comparing acute with acute treatment, maintenance with maintenance and continuation with continuation therapy or relapse prevention with relapse prevention. Furthermore, we only included studies in which the kind of maintenance or continuation therapy was the same as in the acute treatment phase. Since the main goal of this systematic review was to gain information on sustainability, we only included studies reporting sustainability that is a follow-up period of at least 12 months after termination of therapy. We included both, studies reporting sustainability during the follow-up period and sustainability measured after the follow-up period. During the follow-up period, there were no restrictions made with regard to the kind of treatment.

**Table 1:** Inclusion criteria for systematic review.

1) Primary diagnosis: unipolar or chronic depression
2) Age: >18 years old
3) RCT
4) Comparison of psychotherapy and antidepressant pharmacotherapy/psychotherapy and combined treatment/antidepressant pharmacotherapy and combined treatment
5) Comparison of identical treatment phases of the respective treatment methods: Acute psychotherapy and acute antidepressant treatment/maintenance psychotherapy and maintenance antidepressant treatment/recurrence prophylaxis with psychotherapy and recurrence prophylaxis with antidepressants
6) The maintenance treatment/recurrence prophylaxis has to be a continuation of the previous therapy phase
7) Measurement of treatment sustainability during or after a follow-up at least 12 months after treatment termination

Studies with different acute therapies and subsequently the same maintenance therapy (or vice versa) were not included. Otherwise, in the case of long-lasting effects, it is not possible to attribute treatment effects to a certain treatment. Furthermore, studies with comorbid depression and somatic diseases such as cancer and diabetes were excluded.

### Meta-analyses

We conducted independent pairwise meta­analyses for all comparisons between psychotherapy, pharmacotherapy, and combined treatment, using random effects models. To quantify between study heterogeneity, τ^2^ statistics using the Sidik-Jonkman estimator and I^2^ statistics were computed. According to the systematic review, risk ratios (RRs) of relapse, recurrence or rehospitalizations were chosen as the primary outcome and are reported with their 95% CIs. Test statistics and confidence intervals were adjusted by the Hartung and Knapp method. All meta-analytic calculations were conducted using the function metabin of the R-package meta (4.18-0) (22). Forest plots were created using the forest function of the R-package metafor (2.4-0) (23). If not reported in the original study, relapse, recurrence, or rehospitalization rates were calculated as the percentage of participants who experienced the event out of the total number of participants at follow-up. If more than one follow-up assessment was conducted at least 12 months after treatment termination, the longest follow-up interval was chosen as calculation basis.

### Risk of *bias*

UV and BBB independently assessed the included studies concerning their methodological quality according to the Cochrane risk of bias tool by Higgins and Altman (24) (see **Table 2**). We assessed the risk of publication bias using funnel plots for each meta-analysis comparison. Funnel plots were visually inspected for asymmetry to identify potential biases due to missing results. The funnel plots for all comparisons appeared symmetrical, suggesting no significant publication bias. Funnel plots can be found in the **appendix (A2 -A4)**.

**Table 2:** Assessment of risk of bias for included studies according to “The Cochrane Collaboration’s tool for assessing risk of bias in randomized trials” (24).

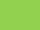 low risk of bias 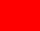 high risk of bias 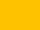 unclear risk of bias	randomization	concealed randomization	blinding of participants/study personnel	data completeness	selective presentations of results	further bias
DeRubeis et al. (25)						
Schaub et al. (26)						
Mergl et al. (27)						
Bausch et al. (28)						
Harkness et al. (29)						
Koppers et al. (30)						
Zobel et al. (31)						
Schramm et al. (32)						
Segal et al. (33)						
Mynors-Wallis et al. (34)						
De Jong-Meyer et al. (35)						
Evans et al. (36)						
Shea et al. (37)						
Miller et al. (38)						
Blackburn et al. (39)						
Simons et al. (40)						
Beck et al. (41)						
Kovacs et al. (42)						
Weissman et al. (43)						

## Results

**Figure 1:**
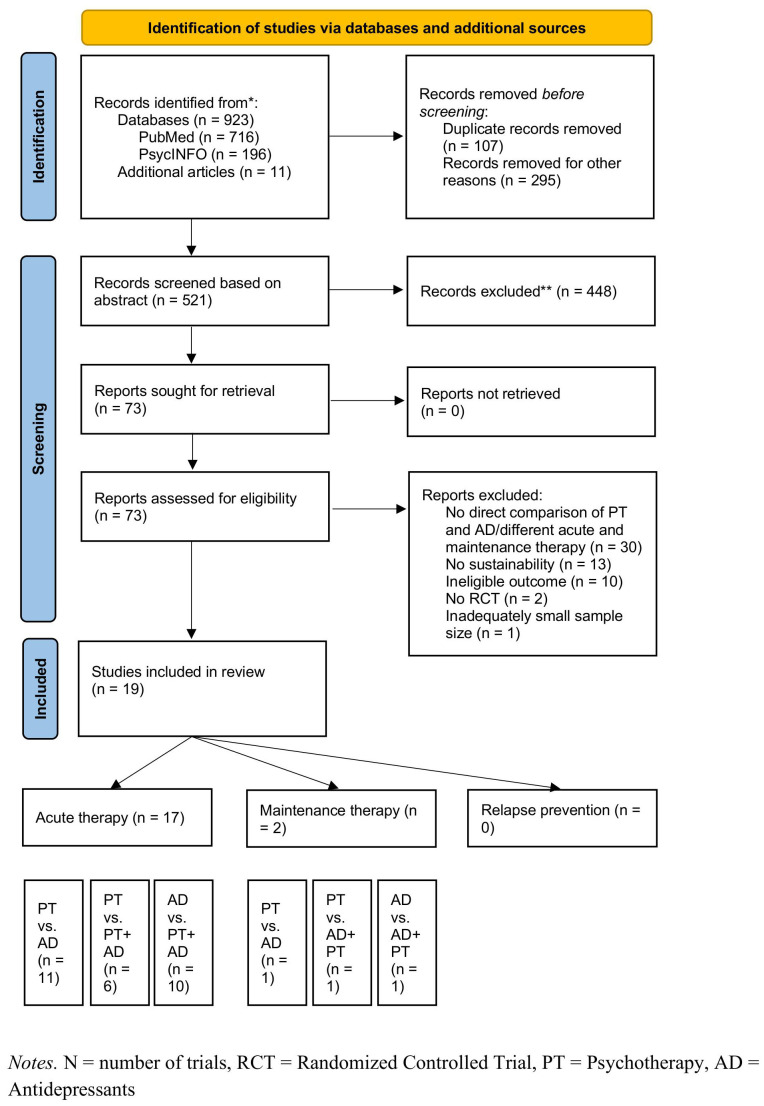
PRISMA flow diagram. N, number of trials; RCT, Randomized Controlled Trial; PT, Psychotheraphy; AD, Antidepresants.

The search yielded a total of 923 studies. Duplicates were removed and the titles and abstracts screened for eligibility by the authors UV, BBB, and MF. Full texts of the identified studies were then carefully read by UV, BBB, and MF to decide on the final inclusion into the systematic review. Relapse, recurrence or rehospitalization rates were chosen as primary outcome. Other potentially relevant outcomes were change in symptom severity, response, or remission. Outcome parameters could be quantified in terms of effect sizes [e.g., Standardized mean difference (SMD), Cohen’s d, Hedge’s g, number needed to treat (NNT)], percentage of relapse/recurrence, or risk of relapse/recurrence [e.g., risk ratio (RR), relative risk ratio (RRR) odds ratio (OR)]. Eventually, 19 RCTs with a mean follow-up period of 23.62 months (range 12-75 months) fulfilled the inclusion criteria (see **Table 1**). The majority of included studies did not contain sufficient information to assess risk of bias as displayed in **Table 2**. In some studies, a high risk of bias needs to be assumed because of using a sequence generated by some rule based on hospital or clinic record number (40), an extreme imbalance in the randomized groups (38), and missing blinding of raters which was likely to have influenced the results (42). In total, 19 studies were included into the qualitative synthesis and 17 studies reported on enduring effects after acute, two studies reported on sustained effects after the end of maintenance therapy (**Figure 1**).

**Figure 2:**
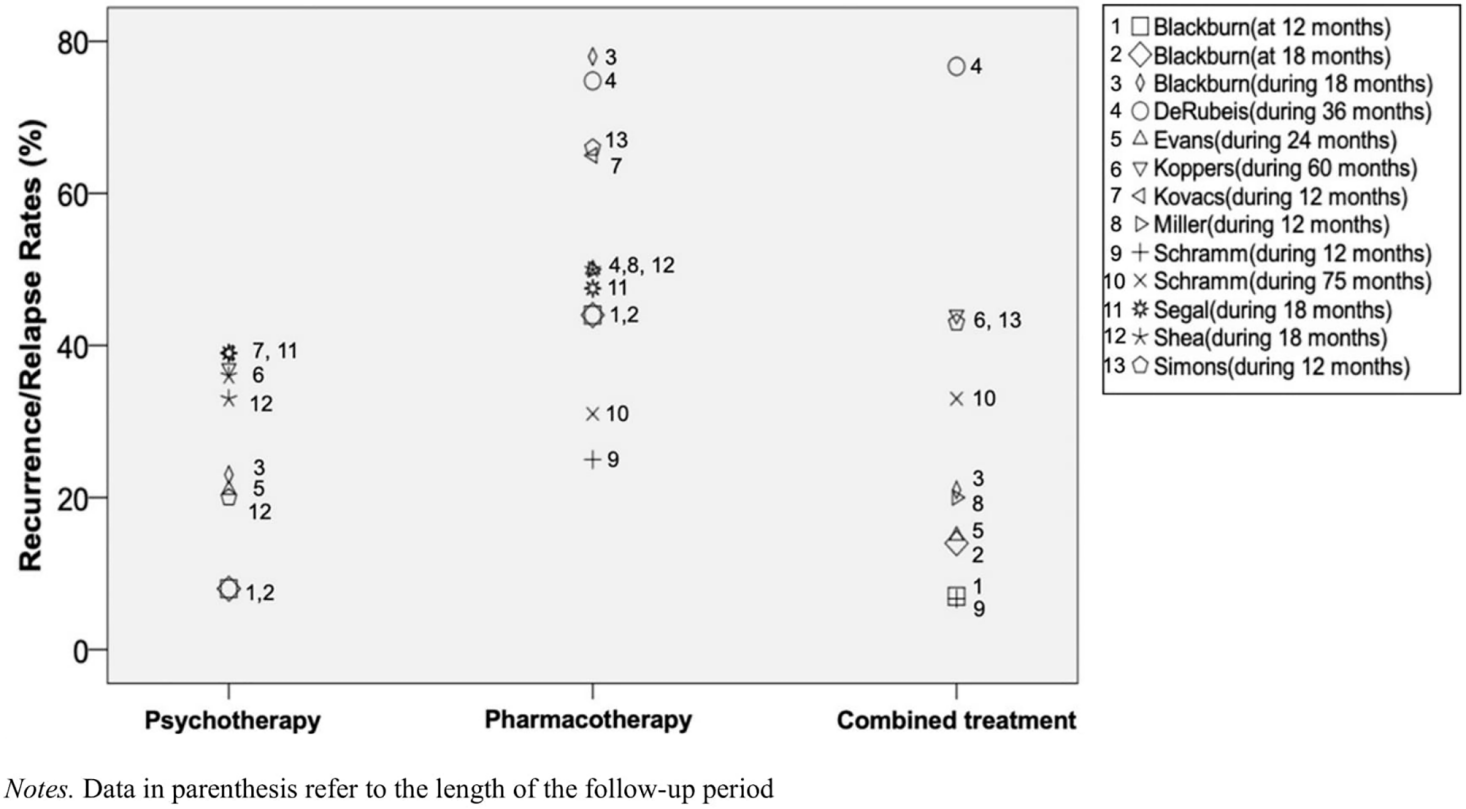
Relapse and recurrence rates between psychotheraphy, pharmacotheraphy, and their combination in depression. Data in parenthesis refer to the length of the follow-up period.

Summarized results of enduring effects of completed acute treatments are displayed in **Table 3** and in-depth information is given in **appendix A5**. Information on additional literature and further descriptive data of included studies can be found in **appendix A6 -7**. **Figure 2** illustrates relapse and recurrence rates in studies with corresponding outcome variables, measured either at a specific follow-up point or during a follow-up period.

**Table 3:** Results of enduring effects of completed acute therapies.

Study	Significant group differences	Outcome
Psychotherapy vs. Antidepressants		
Mergl et al. (27)	n.s.	number of weeks without depressive symptoms
Harkness et al. (29)	n.s.	relapse/recurrence
Segal et al. (33)	n.s.	relapse
Mynors-Wallis et al. (34)	n.s.	remission
Evans et al. (36)	**PT > AD**	**significantly fewer relapses after PT**
Shea et al. (37)	n.s.	recovery and relapse
Simons et al. (40)	n.s.	relapse rate: no significant difference between PT and AD. Patients showed significantly fewer relapses/more frequent remissions after PT and PT+placebo than after AD and combined treatment together.
Kovacs et al. (42)	**PT > AD**	**significant lower BDI scores after PT**
Weissman et al. (43)	n.s.	symptom severity
Bausch et al. (28)	n.s.	sustained response, sustained remission
Psychotherapy vs. Combined treatment		
Koppers et al. (30)	n.s.	recurrence
Mynors-Wallis et al. (34)	n.s.	remission
Evans et al. (36)	n.s.	relapse
Simons et al. (40)	n.s.	relapse rate: no significant difference between PT and combined treatment. Patients showed significantly fewer relapses/more frequent remissions after PT and PT+placebo than after AD and combined treatment together.
Beck et al. (41)	n.s.	remission/recovery
Weissman et al. (43)	n.s.	symptom severity
Combined treatment vs. Antidepressants		
DeRubeis et al. (25)	n.s.	**Recurrence rates, survival rates, sustained recovery**
Schaub et al. (26)	**Combined treatment > AD**	**significantly fewer rehospitalizations after combined treatment**
Zobel et al. (31)	**Combined treatment > AD**	**significantly more frequent persistent remission after combined treatment**
Schramm et al. (32)	**Combined treatment > AD**	**significantly lower symptom severity, more frequent sustained response, more frequent remission after combined treatment**
Mynors-Wallis et al. (34)	n.s.	recovery
De Jong-Meyer et al. (35)	n.s.	response
Evans et al. (36)	**Combined treatment > AD**	**significantly fewer relapses after combined treatment**
Miller et al. (38)	**Combined treatment > AD**	**significantly more frequent remission after combined treatment**
Simons et al. (40)	n.s.	relapse rate: no significant difference between combined treatment and AD. However, patients showed significantly fewer relapses/more frequent remissions after combined treatment, PT and PT + placebo than after AD.
Weissman et al. (43)	n.s.	no significant difference

Studies with significant group differences are boldfaced. PT, Psychotherapy; AD, Antidepressants; n.s., no significant difference, >indicating superiority, <indicating inferiority.

### Enduring effects after discontinued acute treatment by psychotherapy vs. pharmacotherapy (n=11)

#### Sustainability at follow-up (n=4)

Weissman, Klerman (43) found no significant differences in depressive symptoms measured with the Hamilton Rating Scale for Depression (HAMD) between preceding interpersonal therapy (IPT) and antidepressants after 12 months of follow-up. Results by Mynors-Wallis, Gath (34) also showed no significant differences between problem-solving training and antidepressants on recovery rates 13 months after the end of treatment (56-62% vs. 56%). Kovacs, Rush (42) reported significantly lower BDI values after cognitive therapy (CT) compared to drug treatment after 12 months of follow-up. Results by Bausch, Fangmeier (28) yielded no significant differences between pharmacotherapy and CBASP for chronic depression regarding rates of sustained response or sustained remission at a mean follow-up of 4.5 years. Even if not significant, a higher increase in depression scores from post-treatment to follow-up was found for CBASP compared to pharmacotherapy with escitalopram alone.

#### Sustainability during follow-up (n=7)

The studies by Shea, Elkin (37), Segal, Kennedy (33), Harkness, Bagby (29), Mergl, Allgaier (27) revealed no significant differences in relapse rates after prior psychotherapy and antidepressant treatment during a 12- and 18-month follow-up. Despite a 2.1-fold higher probability of relapse after the end of treatment with antidepressants compared to CT during a 12-month follow-up and higher remission rates after CT, the study by Kovacs, Rush (42) showed no significant group differences. Overall, lower, however not significantly, relapse rates and higher remission or recovery rates were observed during the follow-up periods following psychotherapeutic treatments compared to pharmacotherapy (relapse rates for PT: 33-39% vs. AD: 47.5-65%; remission/recovery rates for PT: 26-56% vs. AD: 19-35%). Results from Simons, Murphy (40) showed no significant differences between CT, antidepressants, CT + placebo and CT + antidepressants during a subsequent 12-month follow-up. Preceding treatments without antidepressants (CT and CT + placebo) were significantly superior to antidepressant treatments (antidepressants or antidepressants + CT) in terms of relapse rates (19 vs. 52%) during the follow-up period. Evans, Hollon (36) also found significantly lower rates of relapse within a 24-month follow-up for patients after previous CT than for patients treated with antidepressants (21 vs. 50%).

### Psychotherapy vs. combined treatment (n=6)

#### Sustainability at follow-up (n=3)

After 12 months, no significant differences between IPT and combined treatment could be identified in the study by Weissman, Klerman (43). In another trial by Mynors-Wallis, Gath (34), a comparison of problem-solving training and combined treatment also showed comparable recovery rates after 13 months (56-62 vs. 66%). Beck, Hollon (41) reported a trend toward the superiority of combined treatment compared to CT alone in sustaining depressive symptom reduction after 12 months (82 vs. 58%) although no significant differences were found.

#### Sustainability during follow-up (n=3)

In the study by Koppers, Peen (30), 37% of all patients treated with psychodynamic therapy and 44% of all patients treated with a combination of psychotherapy and pharmacotherapy experienced a relapse over a period of five years; this difference did not reach significance. Evans, Hollon (36) also reported no significant differences in relapse rates after psychotherapy vs. combined treatment over a follow-up period of 24 months (21 vs. 15%). Simons, Murphy (40) found significantly fewer relapses for CT and CT + placebo than for pharmacotherapy alone and in combination with psychotherapy.

### Pharmacotherapy vs. combined treatment (n=10)

#### Sustainability at follow-up (n=5)

Significantly reduced values in BDI and on the HAMD with medium to large effect sizes were reported by Schramm, van Calker (32) at a 12-month follow-up for prior combined inpatient treatment compared to pharmacotherapy alone. Regarding the frequency of sustained response (69 vs. 36%), but not sustained remission (35 vs. 20%), combined treatment was superior to pharmacotherapy. The subsequent study by Zobel, Kech (31) showed no differences between pharmacotherapy and combined treatment on relapse rates (31 vs. 33%) at a 75-month follow-up, but significant differences in terms of sustained remission in favor of combination treatment (11 vs. 28%). Several other studies, e.g. Weissman, Klerman (43), Mynors-Wallis, Gath (34), de Jong-Meyer, Hautzinger (35) did not find significant differences in outcomes between pharmacotherapy alone and combined treatment. However, de Jong-Meyer, Hautzinger (35) Mynors-Wallis, Gath (34) showed that patients achieved response more frequently following combined treatment than following pharmacotherapy alone (64.3/71.4% vs. 33.3%), however without reaching statistical significance.

#### Sustainability during follow-up (n=5)

Evans, Hollon (36) reported a significant higher relapse in patients following treatment with antidepressants than with combination therapy (50 vs. 15%). Although the study by Miller, Norman (38) did not yield significant differences in relapse rates, remission rates after 12 months for prior combined treatment were significantly superior to monotherapy with antidepressants (68% vs. 33%). Schaub, Goldmann (26) reported significantly fewer rehospitalizations (27 vs. 40%) two years after inpatient treatment for a combination of group CBT, pharmacotherapy with AD and clinical management (CM) compared to pharmacotherapy plus CM. The RCT by Schramm, van Calker (32) showed significantly reduced relapse rates in remitted patients over the course of 12 months after combined treatment compared to medication and CM (OR = 0.16, NNT =5, *p*<0.01) during acute inpatient therapy. Relapse rates for pharmacotherapy and for combined treatment did not differ significantly in the RCT by Simons, Murphy (40). However, when the combination of psychotherapy and pharmacotherapy and psychotherapy plus placebo were compared to pharmacotherapy alone, the treatments with psychotherapy resulted in significantly fewer relapses (28 vs. 66%).

### Sustainability after discontinuation of maintenance therapy (n=2)

#### Psychotherapy vs. pharmacotherapy (n=1)

After 12 and 18 months 8% of the participants with prior cognitive maintenance therapy and 44% with prior pharmacotherapeutic maintenance therapy experienced a relapse in the study by Blackburn, Eunson (39). However, this difference was not significant. During the follow-up period, the authors found a significant superiority of a preceding cognitive maintenance therapy versus pharmacotherapeutic maintenance therapy with regard to relapse rates (23 vs. 78%).

#### Psychotherapy/pharmacotherapy vs. combined treatment (n=2)

No significant differences were found between psychotherapy/pharmacotherapy and combined treatment in terms of relapse rates at 12 and 18 months after treatment termination (8/8% and 44/44% vs. 7/14%) in the study of Blackburn, Eunson (39). During the follow-up period of 18 months, relapse rates between psychotherapy and combination treatment were comparable (21 vs. 23%). However, combination treatment resulted in a significantly lower number of relapses compared to pharmacotherapy (21 vs. 78%). A study by DeRubeis, Zajecka (25) yielded comparable results during a 3-year follow-up between antidepressants and CT + antidepressants in terms of recurrence rates (74.8% vs. 76.7%), survival rates (25.2% vs. 23.3%), and rates of sustained recovery (=recovery + no recurrence during FU) (16.6% vs. 17.6%) for chronic or recurrent MDD.

#### Meta-analyses

**Figure 3:**
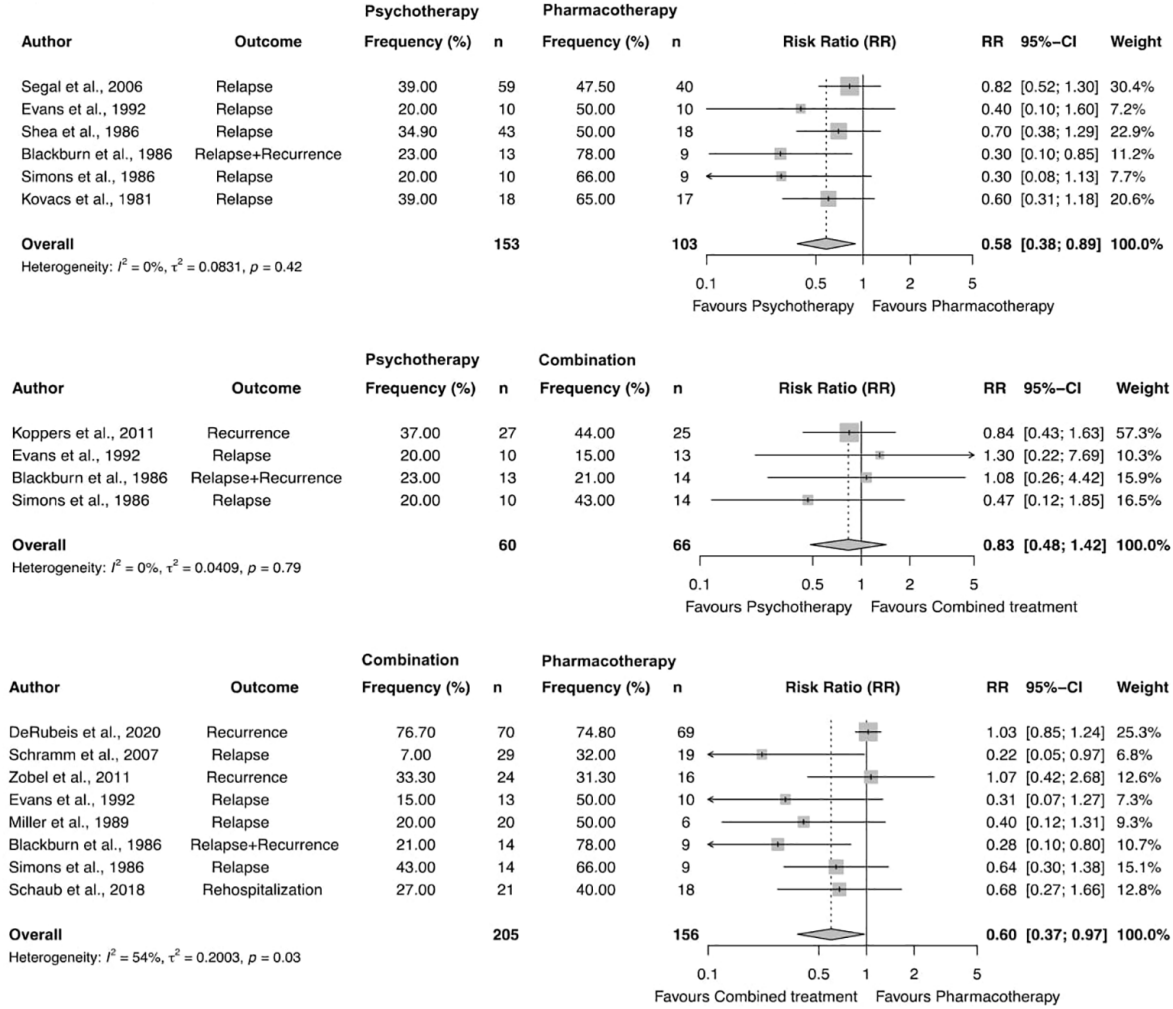
Forest plot and results of pairwise meta-analyses comparing enduring effects of psychotherapy, pharmacotheraphy, and combined treatment in depression.

The pairwise meta-analyses indicated a superiority of combined treatment compared to pharmacotherapy alone in depression in terms of relapse, recurrence and rehospitalization rates (RR=0.60, 95%-CI: 0.42-0.85, p=.011). However, between study heterogeneity was low with I^2^ = 0% (CI: 0.00-70.80%) and τ^2^=.08. Pairwise meta-analyses also yielded that psychotherapy was superior compared to pharmacotherapy alone in terms of relapse, and recurrence rates with RR=0.58 (95%-CI: 0.38-0.89, p=.023) and a low between-study heterogeneity with I^2^ = 0% (95%-CI: 0.00-74.60%) and τ^2^=.08. No significant differences in relapse, and recurrence rates were found for psychotherapy compared to combined treatment (RR=0.83, 95%-CI:0.48-1.42, p=.35). For the comparison of psychotherapy and combined treatment, between-study heterogeneity was low with I^2^ = 0% (CI: 0.00-84.70) and τ^2^=.04. All results are shown in **Figure 3**.

## Discussion

This systematic review and meta-analysis analyzed the comparative sustained efficacy of psychotherapy, antidepressant pharmacotherapy, and their combination in adult patients with depressive disorders. Only RCTs reporting outcomes at least one year after treatment termination, no matter if it was an acute treatment, a maintenance treatment, or a relapse prevention, were included. Combined treatment showed a significantly better long-term outcome considering relapses, recurrence and rehospitalization compared to pharmacotherapy alone, whereas psychotherapy did not differ from combined therapy. The meta-analytic comparison of the limited number of studies indicated a superiority of psychotherapy over pharmacotherapy alone at follow-up. These results are consistent with previous studies showing sustained effects of combined treatment and psychotherapy compared to treatment with antidepressants alone (18, 20, 44–46). A recent network meta-analysis by Furukawa, Shinohara (2) reported some evidence for an advantage of psychotherapies for depression over medications in sustained response after 12 months. In addition, the authors found that the combination treatment but also psychotherapy alone were superior compared to pharmacotherapy alone up to 24 months after the termination of acute treatment. Our results must be interpreted with particular caution for maintenance treatments since only two RCTs could be included.

In choosing a treatment option, besides the efficacy the long-term risk-benefit ratio must be taken into account. While selective Serotonin Reuptake Inhibitors (SSRIs) and selective Serotonin-Noradrenaline Reuptake Inhibitors (SNRIs) show better acceptability than first-generation antidepressants (tricyclic antidepressants) (47), studies in patients taking SSRIs or SNRIs for several years showed a number of critical side effects like weight gain (48), emotional blunting (49), increased sweating (50) libido reduction or sexual dysfunctions (51). Also, both acute and longer lasting withdrawal syndromes including the development of post-withdrawal disorders or alterations of clinical course after antidepressant discontinuation were reported (52, 53). Even though patients must be adequately informed about these possible side effects, Read (54) showed that the patients almost never remembered that they had been educated about possible problems with discontinuation.

An explanation regarding possible negative effects also applies to psychotherapy. While much less investigated, side effects like the emergence of new symptoms, deterioration of existing symptoms, lack of improvement, prolongation of treatment, patient’s non-compliance, strains in the patient-therapist relationship, therapy dependency, suicidality, strains or changes in family or work relations, and risk of stigmatization have been reported (55, 56). Unwanted events are expected to emerge in about 5-20% of all patients treated with psychotherapy, but are often underreported (56). A study by Vaughan, Goldstein (57) showed a 9 to 20 times higher probability to report adverse events in pharmacotherapeutic compared to psychotherapeutic trials. While discontinuation symptoms are described for antidepressants (58), there are lacking data for similar events in psychotherapy. For a more reliable, evidence-based cost-benefit analysis between psychotherapy, pharmacotherapy and their combination, further studies assessing acute and long-term unwanted effects are necessary.

Major limitations of this systematic review and meta-analysis are the overall low number of studies reporting follow-up data after termination of study periods. Concerningly, there were only two RCT reporting on sustainability after maintenance treatment and even none reporting on sustainability after relapse prevention. Also, none of the included studies were without any concern for a risk of bias. Further limitations were heterogeneity in patients’ duration of illness, times of measurement (during vs. at follow-up), and inclusion criteria e.g., if only responders were included. In particular, the latter approach seems critical, since the onset of treatment effects may be delayed, especially in studies with psychotherapy. While we only included studies reporting relapse, recurrence or rehospitalization as well as change in symptom severity, response, or remission, we did not have a specific definition of these terms as an inclusion criterion. Therefore, there may be heterogeneity in the definition of outcomes.

When interpreting the results it is also important to account for the fact that different self-rated and observer-rated instruments were used for the definition of the outcome and thus measuring different aspects of depression as discussed by Fried, Flake (59). Other methodologically limiting factors are the naturalistic follow-up in all studies as well as the disregard of symptom severity as an influencing factor in treatment outcome. Further patient characteristics such as demographics and symptoms of depression are factors influencing each study outcome and were not considered in this analysis. Additionally, as some of the included studies are from the 1980’s the question arises whether psychotherapeutic treatments from 40 years ago are comparable to current standards.

Future studies should consider that treatment efficacy may differ depending on symptom course or prior response to treatment (60, 61). Furthermore, choosing adequate outcome variables to assess treatment effects is of great importance. Next to symptom improvement, recovery, response, relapse and recurrence, social functioning and quality of life should be considered. In addition, RCTs on long-term effects of internet-based treatment formats and of sequenced treatments are still lacking. A study on sequenced treatments by Guidi and Fava (21) indicated for example that the combination of psycho- and pharmacotherapy after response to acute-phase pharmacotherapy was superior to active control conditions in reducing relapse/recurrence risk, while subsequent psychotherapy without medication was equivalent to active control conditions (e.g., antidepressant medication).

Since depression is considered a recurrent disorder, enduring treatment effects are of high importance. Our results provide evidence that patients are more likely to experience sustained effects after a follow-up time after the termination of a combination of psychotherapy (regardless specific approaches) and antidepressants or psychotherapy compared to antidepressants alone. The results also suggest that combined treatment is not superior to psychotherapy alone in the long-term. Further studies on long-term sustained treatment effects and long-term side effects would be necessary in view of the chronicity and recurrent nature of depressive disorders. Future practice guidelines and participatory decision-making processes for the choice of a form of therapy should take into account the current knowledge on long-term sustained effects of antidepressant therapy methods more than has been the case to date.

## Data Availability

The original contributions presented in the study are included in the article/**Supplementary Material**. Further inquiries can be directed to the corresponding author.
