# Supplementary material for: Enduring effects of psychotherapy, antidepressants and their combination for depression: a systematic review and meta-analysis
Source: Front Psychiatry. 2024 Nov 27;15:1415905. doi: 10.3389/fpsyt.2024.1415905 (PMC11632389; doi:10.3389/fpsyt.2024.1415905)
Supplement: Supplementary file 1 [file DataSheet1.zip › Appendix 1.DOCX]

**A1 Keywords for literature research:** (depressive [title] OR depression [title] OR depressed [title] OR "affective disorder" [title] OR “major depressive disorder” [title] OR MDD [title]) AND ("long-term" OR "enduring effect" OR "lasting effect" OR "persist" OR relapse OR maintenance OR stability OR stable OR recurrence OR continuation) AND (“follow-up”) AND (CBT OR “cognitive behavioral therapy” OR “cognitive behavioural therapy” OR “CT” OR “cognitive therapy” OR IPT OR “interpersonal therapy” OR MBCT OR “mindfulness based cognitive therapy” OR ACT OR “acceptance and commitment therapy” OR MBSR OR “mindfulness based stress reduction” OR psychodynamic OR psychoanalysis OR psychoanalytic OR psychotherapy OR psychotherapeutic) AND (antidepressant OR SSRI OR “selective serotonin reuptake inhibitors” OR SNRI OR “serotonin-norepinephrine reuptake inhibitors” OR “MAO inhibitors” OR “monoamine oxidase inhibitors” OR NARI OR “noradrenaline reuptake inhibitors” OR “tricyclic antidepressants” OR TCA OR “tetracyclic antidepressants” OR TeCA OR pharmacotherapy) AND (randomized OR randomization OR RCT OR control OR controlled OR “control group” OR "randomized controlled trial")
