# Supplementary material for: Enduring effects of psychotherapy, antidepressants and their combination for depression: a systematic review and meta-analysis
Source: Front Psychiatry. 2024 Nov 27;15:1415905. doi: 10.3389/fpsyt.2024.1415905 (PMC11632389; doi:10.3389/fpsyt.2024.1415905)
Supplement: Supplementary file 1 [file DataSheet1.zip › Appendix 5.DOCX]

A5. Summary of included studies.

| Study | N* | dp/i/o | C | Comparison | Inclusion criteria | Outcome | A/M | Psychotherapy – Interventions and duration | Pharmacotherapy – Interventions and duration | Short-term outcome | Definition of treatment effects at follow-up | Inclusion in final analysis of sustainability | Follow-Up (months) | Time interval without treatment for depression | Long-term outcome |
| --- | --- | --- | --- | --- | --- | --- | --- | --- | --- | --- | --- | --- | --- | --- | --- |
| DeRubeis et al., 2020  Preceding study: Hollon et al., 2014 | 452 | o | USA | CT + AD vs. AD | Age: >=18;  HAMD>=14  chronic MDD (episode duration ≥ 2 years) or recurrent MDD (an episode in the past 3 years even if only a second episode) using the DSM-IV  Only eligible for FU, if recovery was previously achieved | LIFE > 5 for 2 consecutive weeks | A/M | CT: 2 sessions of 50 minutes per week for the first 2 weeks, then 1 session of 50 minutes per week for acute treatment;  1 session of 50 minutes per months for continuation up to 42 months | Multiple trials with SSRI or SNRI; if necessary switched to tricyclic AD or MAO  Stable medication during continuation  Medical visits weekly for the first month, then biweekly and monthly for continuation up top 42 months | **Remission rates: CT + AD = AD**  **Recovery rates: CT + AD > AD** | Recurrence rates | **N = 139** | 36 | CT: 36 months  AD: slowly tapering without specified interval | **During FU**  **Recurrence rates:**  **CT + AD: 74.8%**  **AD: 76.7%**  **CT + AD = AD** |
| Schaub et al., 2018 | 177 | i | G | Group CBT + AD + CM vs. GroupCBT + individual CBT + AD + CM vs. AD + CM | Age: 18-69;  depression according to DSM-IV; remitted acute symptoms | HAMD  BDI | A | Group CBT: 2 sessions of 90 minutes per week over 8 weeks group Group CBT + individual CBT: Group CBT plus 16 individual sessions in outpatient setting (25-50 minutes over 6 months) | All patients received drug treatment for the duration of their hospital stay; mostly AD | **Group CBT + AD =Group -CBT + individual CBT + AD = AD** | number of rehospitalizations | **N = 69** | 24 | Not specified | **During FU**  Number of rehospitalizations:  Group CBT + AD + CM: 27%  Group CBT+ individual CBT + AD + CM: 34%  AD + CM: 40%  **Group CBT + AD > AD**  **Group CBT + individual CBT = AD** |
| Bausch et al., 2017  Preceding study: Schramm et al., 2015 | 59 | o | G | CBASP vs. AD + CM vs. CBASP + AD (augmented cases who did not improve after 8 weeks of CBASP or AD ) | Age: 18-65  MADRS ≥28  Medication free for at least 2 weeks before the study started;  chronic MDD, however, modified by at least one year of depressive symptomatology or recurrent MDD episodes with ≥3 episodes; the preceding episode had to be within the last 2.5 years | IDS-SR,  MADRS | A | 12 individual sessions of CBASP | Escitalopram 10- 20 mg/day; zolpidem and pipamperone were allowed up to 3 weeks; CM were weekly meetings up to 20 minutes | **Significant Improvement:**  **CBASP = AD + CM**  **Response:**  **CBASP: 68.4%**  **CBASP + AD: 45%**  **AD: 60%**  **Remission:**  **CBASP: 36.8%**  **CBASP + AD (augmented): 30%**  **AD: 50%** | Significant improvement measured with the IDS-SR | 43 | 54 | Whole sample: 93% received AD, 45% received AD + psychotherapy  AD: 31% were still taking the medication, 41% were taking another AD | **At FU:**  **CBASP = AD = CBASP+AD (augmented)** |
| Mergl et al., 2018  Preceding study: Hegerl et al., 2010 | 368 | o | G | CBT vs. AD vs. PL vs. self-help vs. Patients‘ Choice Arm (AD vs. CBT) | Age: ≥ 18;  8 ≤HAMD ≤22;  Dysthymia; mild to moderate depression; Patients were not allowed to receive PT or take AD currently | PSR | A | CBT, individual sessions of 50 minutes,  9 group sessions of 90 minutes with 5-8 participants over 10 weeks  82 patients could freely choose between PT and AD (uncontrolled treatment condition) | Sertraline (increased from 50 mg to 200 mg per day),  medical visits in week 1 ,2, 4 ,6 , 8 and 10 | **AD = CBT** | Number of weeks without depressive symptoms -> PSR = 1 - 2 | Completer of acute therapy; only patients who either received no further therapy during FU or a continuation of the acute therapy without further treatment were included,  only data from the AD and CBT condition and also freely chosen treatment condition included,  N=77 | 12 | AD: n = 15 continuation therapy with AD, n = 12 no treatment  CBT: n =5 continuation therapy with CBT, n =14 no treatment  Patients‘ Choice condition:  AD: n = 12 continuation therapy with AD, n = 5 no treatment  CBT: n= 4 months for continuation therapy with CBT, n = 10 no treatment | **During FU**  Absence of symptoms  *p* = 0.4 (n.s.)  **CBT = AD** |
| Harkness et al., 2012  Preceding study: McBride et al., 2006 | 203 | o | CA | CBT vs. IPT vs. AD | Age: 18-60;  Depression according to DSM-IV;  HAMD ≥ 16  🡪 moderate to severe depression;  Patients were not allowed to take AD | HAMD  SCID I | A | IPT or CBT over 16 weeks,  1 session per week | Duration of 16 weeks, one of the following drugs used: bupropion, citalopram, fluoxetine, fluvoxamine, phenelzine, sertraline, venlafaxine; medical visits every second week | Response Rates:  CBT: 60%  IPT: 54%  AD: 72%  **CBT = IPT =AD**  **CAVE:** in patients who experienced child abuse ->  **IPT < CBT=AD** | relapse/recurrence: HAMD > 15 and depression (SCID-I) in the first four months/more than four months | Completer**: N = 65**  ITT: **N = 94** | 12 | naturalistic | **During FU**  relapse/recurrence:  Completer: *p* = .80 (n.s.)  ITT: *p* = .97 (n.s.)  **CBT = IPT = AD**  **CAVE:** Child abuse as a significant predictor of relapse/recurrence. OR: 2.89 |
| Koppers et al., 2011  Preceding study: de Jonghe et al., 2004 | 106 | o | NL | PDT vs. PDT + AD | Age: 19-65;  Depression according to DSM-IV; mild/moderate depression | HAMD  CIDI | A | PDT, 16 sessions over 6 months | Starting medication: Venlafaxine; change to other SSRIs (especially fluvoxamine), TCA (nortryptiline), nortryptiline + lithium possible; duration of 6 months | Recovered:  PDT: 32,1%;  PDT + AD: 42,4%  **PDT + AD= PDT** | Recurrence: CIDI | Completer: **N = 52** | 60 | naturalistic | **During FU**  Recurrence:  PDT: 37%, PDT + AD: 44%  **PDT = PDT + AD** |
| Zobel et al., 2011  Preceding publication: Schramm et al., 2007 | 124 | i | G | IPT + AD vs. AD + CM | Age: 18-65;  MDD according to DSM-IV; HAMD ≥16  🡪 moderate to severe depression | HAMD  BDI | A | IPT (15 individual and 8 group sessions, involvement of family members possible), duration of 5 weeks | Starting medication:  Sertraline;  ITT: 80.2 mg/day (SD 32.9 mg/day);  Completer: 77.5 mg/day (SD 31.3 mg/day)  Possible change: Amitriptyline / Amitriptyline oxide; ITT: 160.8 mg/day (SD 58.2 mg/day); Completer: 167 mg/day (SD 63.4 mg/day) | ITT:  BDI: **IPT + AD = AD + CM**  HAMD: **IPT +** **AD > AD + CM**  Response (HAMD):  70% (IPT + AD);  51% (AD + CM)  **IPT + AD > AD + CM**  Remission:  49% (IPT + AD);  34% (AD + CM)  **IPT + AD = AD +CM** | Response:  At least 50% improvement in HAMD  Remission:  HAMD ≤ 7  Relapse  HAMD ≥ 15 + psychiatric status ratings score ≥ 5 for min. of 2 weeks  Sustained Response:  At least 50% improvement on HAMD for post measurement and nor relapse/hospitalizatio nduring FU  Sustained Remission:  HAMD ≤ 7 and PSR 1-2 for at least 2 weeks after achieving the initial remission | Completer FU 1: N=97  ITT  FU2: N=97 | FU1:  12  FU2:  75 | naturalistic,  FU1: 98% received further treatment, no significant differences between groups; At 12 months: 17% discontinued from any form of therapy  FU2: 60% used AD, 19% received PT; during FU2 56% received at least one PT treatment, 80% used AD; 37% were at least once rehospitalized; no difference between groups | At FU1:  Relapse of former responders:  IPT + AD: 13%  AD + CM: 39%  **IPT + AD = AD +CM**  Relapse of former remitters  IPT + AD: 7%  AD + CM: 32%  **IPT + AD > AD +CM**  Sustained response:  IPT + AD: 69%  AD + CM: 36%  **IPT + AD > AD +CM**  Sustained remission:  IPT + AD: 35%  AD + CM: 20%  **IPT + AD = AD +CM**  During FU2:  Recurrence of MDD:  IPT + AD: 33%  AD + CM: 31%  **IPT + AD = AD +CM**  At FU2:  IPT + AD: 28%  AD + CM: 11%  **IPT + AD > AD +CM** |
| Segal et al., 2006 | 301 | o | Ca | BT vs. AD | Age: 18-65;  Depression according to DSM-IV; HAMD >12  🡪 mild to severe depression | HAMD | A | BT, 20 individual sessions of 50 minutes, duration of 22-24 weeks | Paroxetine 20-50 mg or Sertraline 50-200 mg or Venlafaxine 75-225mg, 10-13 medical visits, 6 months | 127 Completer,  Response rate 80% for AD, 72 % for PT,  Remission rate 71 % for AD, 61% for PT  **No statement on superiority of treatments** | Relapse:  BDI >=15 OR HAMD>=16  and depression according to LIFE | Completer: **N=78** | 18 | naturalistic  AD: 86% with medication for maintenance | **During FU**  Relapse:  PT: 39 %, AD: 47.5% (n.s.)  **CT = AD** |
| Mynors-Wallis et al., 2000 | 151 | o | UK | PL (Nurse) vs. PL (Physician) vs. AD vs. AD + PL (Nurse and Physician) | Age: 18-65  depression according to RDC  HAMD >13  🡪 mild to severe depression; at least 1 month no AD, currently no PT/AD | HAMD | A | PL, 6 individual sessions over 12 weeks | Fluvoxamine (initial: 100 mg/day), Paroxetine (initial: 20 mg/day), 6 medical visits, 12 weeks | **PL = AD = AD + PL** | Recovery:  HAMD ≤ 7 | Only Completer: **N=113** | 12 | naturalistic | **At FU**  Recovery:  PL physician: 62%  PL nurse: 56%, AD: 56%, PL + AD: 66%  **PL = AD = AD + PL** |
| De Jong-Meyer et al., 1996 | 155 | i/o | G | CBT + AD vs. AD + SC | Age: 40-60;  endogenous depression according to ICD-9 and DSM-II; BDI/HAMD > 20; Only personality disorders as possible comorbidity; wash-out period of 7 days | BDI, HAMD | A | CBT, 3 sessions 20-30 minutes per week, duration of 8 weeks | Amitriptyline (150mg/day),  + 3 sessions of 20-30 minutes per week with supportive care, duration of 8 weeks | BDI/HAMD ≤ 9  Response rate:  AD + SC (o): 25/35 %  AD +SC (i): 51/55%  AD +BT (o): 36/67%  AD + BT (i): 44/56  i > o  **CBT + AD = AD +SC** | Response:  BDI/HAMD ≤ 9 | ITT: **N = 155** | 12 | naturalistic | **At FU**  Response:  AD + SC (o):33/33%  AD + SC (i): 69/67%  AD + BT (o): 64/71%  AD + BT (i): 56/68%  i > o  **CBT + AD = AD + SC** |
| Evans. et al., 1992  Preceding study:  Hollon et al., 1992 | 107 | o | USA | CT vs. AD vs. AD + CT | Age: 18-62; Major Depression according to RDC | BDI, HAMD | PT: A  AD: A, M | CT, maximum of 20 sessions, duration of 12 weeks | Imipramine hydrochloride (75-300 mg/day), duration of 12 weeks, Maintenance therapy for 12 months | **CT = AD = CT + AD**  (Hollon et al., 1992) | Relapse: BDI ≥ 16 for two consecutive weeks | Only patients who achieved response (BDI ≤15) and Completer:  **N = 44** | 24 (A)  12 (M) | naturalistic  Patients were instructed to avoid any further treatment during FU | **During FU**  Relapse:  AD_A_: 50%  CT + AD: 15%  CT: 18 %  AD_M_: 32%  **CT = AD_E_= CT + AD > AD** |
| Shea et al., 1992  Preceding studies: Elkin et al., 1985; Elkin et al., 1989 | 250 | o | USA | CBT vs. IPT vs. AD + CM vs. PL + CM | Age: 21-60; Major Depression according to RDC;  HAMD ≥14 | LIFE-II PSR, HAMD | A | CBT, IPT  16-20 sessions of 50 minutes, duration of 16 weeks | imipramine hydrochloride week 4: 163 mg/day), week 8: 231 mg/day,  16-20 sessions (first one 45-60 minutes, then 20-30 minutes), duration of 16 weeks | **CBT = IPT = AD + CM =**  **PL + CM** | Recovery: LIFE-II, PSR= 1-2 for at least eight consecutive weeks  Relapse: PSR= 5 or 6 (RDC) for two consecutive weeks or treatment of depression for three consecutive weeks | Only patients who achieved recovery after acute therapy: **N=47** | 18 | naturalistic Treatment during FU: CBT: 14%, IPT: 43%, AD+CM: 44%, PL + CM: 27% | **During FU**  Recovery without relapse:  CBT: 30%  IPT: 26%  AD + CM: 19%  PL + CM: 20%  Relapse:  CBT: 36%  IPT: 33%  AD + CM: 50%  PL + CM: 33%  Relapse in patients with recovery after acute therapy and without during the first eight weeks:  CBT: 9%  IPT: 24%  AD + CM: 28%  PL + CM: 25%  Relapse in patients with recovery after acute therapy:  CBT: 39%  IPT: 56%  AD + CM: 45%  PL + CM: 42%  **CBT = IPT = AD + CM = PL + CM** |
| Miller et al., 1989 | 45 | i/o | USA | AD vs. AD + CT vs. AD + SST | Age: 18-65; Major Depression according to Diagnostic Interview Schedule; BDI/HAMD > 17 | HAMD, BDI | A | Inpatient CT  + outpatient CT for 4 months; daily sessions for inpatient treatment, weekly sessions for outpatient treatment | inpatient + 4 months  amitriptyline or desipramine at least 150 mg/day, further medication allowed if indicated | recovery:  CT: 87% (Completer), 71% (ITT)  AD: 60% (Completer), 35% (ITT)  **AD + CT = AD + SST > AD** | Recovery: HAMD < 7, BDI < 9, Suicidal Ideation < 7  Relapse: achieved recovery  + subsequent  1) HAMD > 17  2) BDI > 16  3) Suicidal Ideation > 7  4) Rehabilitation | Responder and Non-Responder: **N = 33** | 12 | naturalistic  ¾ (73 % of CT, 78% of AD) received further outpatient treatment during FU; no difference for number of rehospitalizations (CT: 14%, AD: 22%); | **During FU**  Relapses tend to be more frequent in AD than in CT+AD (50% vs. 20%);    Remission more frequent in CT + AD vs. AD (68% vs. 33%)  ITT: 54% vs. 18%  **CT + AD = AD (relapse)**  **CT + AD > AD (remission)** |
| Blackburn et al., 1986  Preceding studies:  Blackburnet al., 1981, Blackburn and Bishop., 1983 | 88 | dp/o | UK | CT vs. AD vs. CT +AD | Age: 18-65; Major Depression according to PSE and RDC; BDI >= 14 | BDI, HAMD | M | CT, 12-–15 weeks  Following acute therapy: booster sessions every 6th week over 6 months | No regulations, mostly amitriptyline or clomipramine (150 mg/day); 12-15 weeks, 6 months of maintenance therapy | outpatients:  **CT + AD = CT > AD**  day patients:  **CT + AD > CT = AD**  (Blackburn and Bishop, 1983; Blackburn et al., 1981) | Response: BDI <=8 and/or HAMD<=9  relapse: assessment by physicians regarding reoccurrence of depression | Responder: **N=41** | 12 and 18 | naturalistic | **At FU**  Recurrence: 12/18 months  CT: 8/8%  AD: 44/44%  CT + AD: 7/ 14%  n.s.  **-> CT = AD = CT + AD**  **During FU**  relapse after 18 months:  CT: 23%  AD: 78%  CT + AD: 21%  -> **CT = CT+AD > AD** |
| Simons. et al., 1986  Preceding study:  Murphy et al., 1984 | 87 | o | USA | CT vs. AD vs. CT + PL vs. CT + AD | Age 18-60; National Institute of Mental Health Diagnostic Interview Schedule; BDI ≥20; HAMD ≥14 | BDI, HAMD | A | CT, maximum of 20 sessions of 50 minutes, duration of 12 weeks | Nortriptyline + maximum of 12 sessions of CM, duration of 12 weeks | **CT = AD = CT + PL= CT + AD**  (Murphy et al., 1984) | Non-Responder: BDI ≥10  Responder: BDI <10  relapse/recurrence: BDI>=16 or new treatment due to depression | Responder: **N = 44** | 12 | naturalistic  53% received further treatment during FU or experienced a relapse: 81% among non-Responders and 36% among tesponders | **During FU**  Relapse:  AD: 66%  CT: 20%  CT + PL: 18%  CT + AD: 43%  CT + PL + CT + AD: 28 %  CT + CT + PL: 19%  AD + AD + CT: 52%  **CT = AD = CT + PL = CT + AD**  **CT + CT + PL > AD + AD + CT**  **CT + PL + CT + AD > AD**  Remission:  **CT + PL + CT + AD > AD** |
| Beck et al., 1985 | 33 | o | USA | CT vs CT + AD | Age: 20-65; depressive syndrome according to Feigner's criterion; BDI ≥20; HAMD >= 14 | BDI, HAMD | A | CT, 20 sessions over 12 weeks | Amitriptyline hydrochloride  (75-200 mg/day); duration of 12 weeks | n.s., CT tends to be better than CT + AD (71% remission vs. 45% partial Response)  **CT = CT + AD** | markedly or completely improved:  BDI = 0 - 9 | Completer: **N = 22** | 12 | naturalistic  CT + AD: 91 % received further sessions of CT (M = 14.2);  CT: 71% received further sessions of CT (M = 5.9) | **At FU**  markedly or completely improved:  CT: 58%, CT + AD: 82%  n.s.  **CT = CT + AD** |
| Kovacs et al., 1981  Preceding study:  Rush et al., 1977 | 41 | o | USA | CT vs. AD | Age: 18-65; HAMD ≥ 14; BDI ≥20 | BDI, HAMD | A | CT, maximum of 20 sessions in 12 weeks | Imipramine hydrochloride  (75--250 mg/day) + maximum of 12 sessions of CM of 20 minutes; duration of 12 weeks | **CT > AD**  (Rush et al., 1977) | Remission: BDI < 16 | Completer: **N = 35** | 12 | naturalistic  AD: 5 for further PT,  6 for AD  CT: 5 for further PT,  2 for AD | **At FU:**  BDI ≤ 9:  CT: 67%  AD: 35% (n.s.)  **CT = AD**  BDI values significantly lower for CT than for AD  **CT > AD**  **During FU**  Remission:  CT: 56 %  AD: 35 % (n.s.)  Relapse (BDI)  CT: 39%  AD: 65% (n.s.)  **CT = AD** |

*Notes.* A = acute treatment, AD = antidepressants, BDI = Beck depression inventory, BT = behavioural therapy, C = Country, CA = Canada, CIDI = Composite International Diagnostic Interview, CBT = cognitive behavioural therapy, CBASP = cognitive behavioral analysis system of psychotherapy, CT = icognitive therapy, CM = clinical management, dp = day clinic, FU = follow-up, G = Germany, HAMD = Hamilton depression scale, i = inpatient, IDS-SR = Inventory of Depressive Symptomatology, ITT = intention to treat, IPT = interpersonal psychotherapy, LIFE II-II = Longitudinal Interval Follow-up Evaluation, M = maintenance treatment, MADRS = Montgomery-Åsberg Depression Rating Scale, MAO = monoamine oxidase inhibitor, MDD = major depressive disorder, N* = randomized sample size at baseline, N = sample size, n.s. = not significant, NL = Netherlands, o = outpatient, OR = odds ratio, PT = psychotherapy, PDT = psychodynamic psychotherapy, PSR = psychiatric status rating, PL = placebo, RDC = research diagnostic criteria, SC = supportive care, SD = standard deviation, SNRI = Selective Serotonin-Noradrenaline-Reuptake-Inhibitor, SSRI = Selective Serotonin-Noradrenaline-Inhibitor, SST = social skills training, USA = United States of America, UK = United Kingdom
