# Supplementary material for: Enduring effects of psychotherapy, antidepressants and their combination for depression: a systematic review and meta-analysis
Source: Front Psychiatry. 2024 Nov 27;15:1415905. doi: 10.3389/fpsyt.2024.1415905 (PMC11632389; doi:10.3389/fpsyt.2024.1415905)
Supplement: Supplementary file 1 [file DataSheet1.zip › Appendix 6.DOCX]

A6 Additional literature:

**References of preceding studies**

Blackburn, I. M., & Bishop, S. (1983). Changes in cognition with pharmacotherapy and cognitive therapy. *British Journal of Psychiatry, 143*, 609-617. doi:10.1192/bjp.143.6.609

Blackburn, I. M., Bishop, S., Glen, A. I., Whalley, L. J., & Christie, J. E. (1981). The efficacy of cognitive therapy in depression: a treatment trial using cognitive therapy and pharmacotherapy, each alone and in combination. *Br J Psychiatry, 139*, 181-189.

de Jonghe, F., Hendricksen, M., van Aalst, G., Kool, S., Peen, V., Van, R., van den Eijnden, E., & Dekker, J. (2004). Psychotherapy alone and combined with pharmacotherapy in the treatment of depression. *Br J Psychiatry, 185*, 37-45.

Elkin, I., Parloff, M. B., Hadley, S. W., & Autry, J. H. (1985). NIMH treatment of Depression Collaborative Research Program: Background and research plan. *Archives of General Psychiatry, 42*(3), 305-316. doi:10.1001/archpsyc.1985.01790260103013

Elkin, I., Shea, M., Watkins, J., Imber, S., Sotsky, S., Collins, J., Glass, D. R., Pilkonis, P. A., Leber, W. R., Docherty, J. P., Fiester, S. J., & Parloff, M. B. (1989). National Institute of Mental Health Treatment of Depression Collaborative Research Program. General effectiveness of treatments. *Arch Gen Psychiatry, 46*(11), 971-982; discussion 983.

Hegerl, U., Hautzinger, M., Mergl, R., Kohnen, R., Schutze, M., Scheunemann, W., Allgaier, A. K., Coyne, J., & Henkel, V. (2010). Effects of pharmacotherapy and psychotherapy in depressed primary-care patients: a randomized, controlled trial including a patients' choice arm. *Int J Neuropsychopharmacol, 13*(1), 31-44. doi:10.1017/s1461145709000224

Hollon, S. D., DeRubeis, R. J., Evans, M. D., Wiemer, M. J., Garvey, M. J., Grove, W. M., & Tuason, V. B. (1992). Cognitive therapy and pharmacotherapy for depression. Singly and in combination. *Arch Gen Psychiatry, 49*(10), 774-781.

McBride, C., Atkinson, L., Quilty, L. C., & Bagby, R. M. (2006). Attachment as moderator of treatment outcome in major depression: a randomized control trial of interpersonal psychotherapy versus cognitive behavior therapy. *J Consult Clin Psychol, 74*(6), 1041-1054. doi:10.1037/0022-006x.74.6.1041

Murphy, G., Simons, A., Wetzel, R., & Lustman, P. (1984). Cognitive therapy and pharmacotherapy. Singly and together in the treatment of depression. *Arch Gen Psychiatry, 41*(1), 33-41.

Rush, A., Beck, A., Kovacs, M., & Hollon, S. (1977). Comparative efficacy of cognitive therapy and pharmacotherapy in the treatment of depressed outpatients. *Cognitive Therapy and Research, 1*(1), 17-37. doi:10.1007/bf01173502

Weissman, M., Prusoff, B., Dimascio, A., Neu, C., Goklaney, M., & Klerman, G. (1979). The efficacy of drugs and psychotherapy in the treatment of acute depressive episodes. *Am J Psychiatry, 136*(4B), 555-558.
