# Supplementary material for: Enduring effects of psychotherapy, antidepressants and their combination for depression: a systematic review and meta-analysis
Source: Front Psychiatry. 2024 Nov 27;15:1415905. doi: 10.3389/fpsyt.2024.1415905 (PMC11632389; doi:10.3389/fpsyt.2024.1415905)
Supplement: Supplementary file 1 [file DataSheet1.zip › Appendix 7.DOCX]

| A7 Overview of countries, where included studies were conducted and of psychotherapy methods and antidepressants | |
| --- | --- |
|  |  |
|  |  |
|  |  |
| *Note:* The listed drugs represent the initial medication in included studies; due to missing information on the exact initial medication by Schaub et al. (2018), the information on antidepressants is not shown here. | |
