# Supplementary figures and images for: Enduring effects of psychotherapy, antidepressants and their combination for depression: a systematic review and meta-analysis
Source: Front Psychiatry. 2024 Nov 27;15:1415905. doi: 10.3389/fpsyt.2024.1415905 (PMC11632389; doi:10.3389/fpsyt.2024.1415905)

A2 Funnel Plot combination treatment vs pharmacotherapy


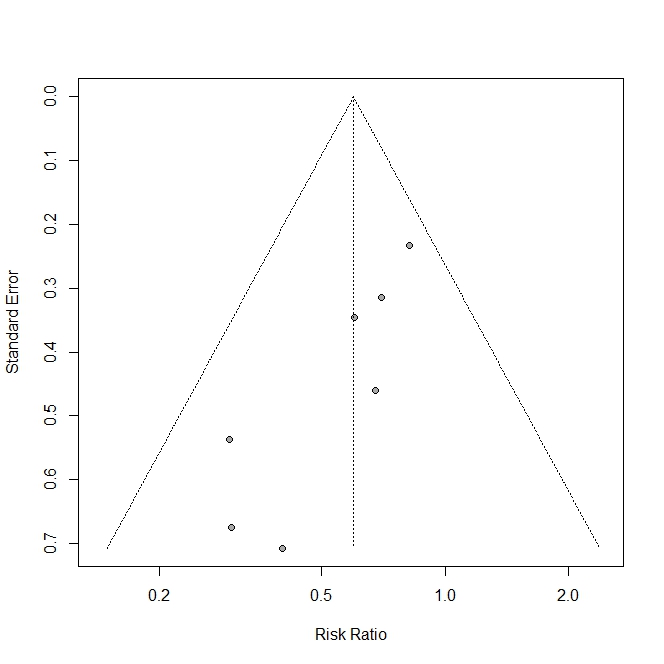

Supplement: Supplementary file 1 [file DataSheet1.zip › Appendix 2.DOCX]

A3 Funnel Plot psychotherapy vs combination treatment
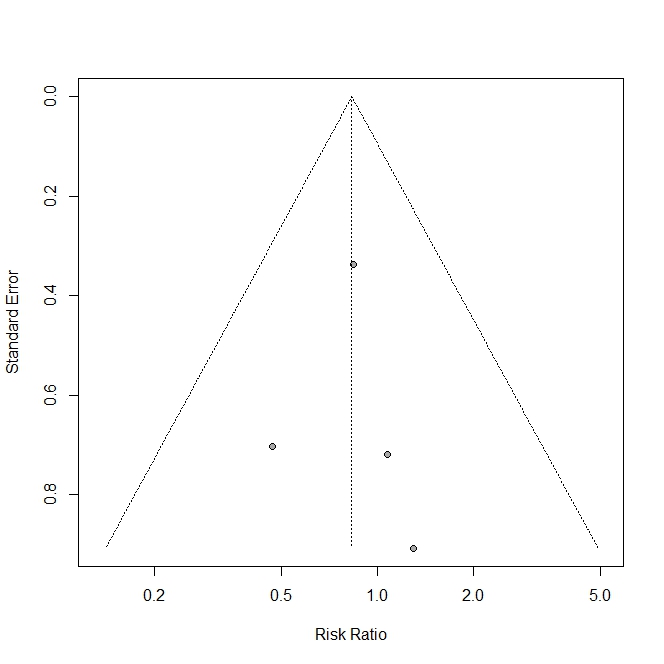

Supplement: Supplementary file 1 [file DataSheet1.zip › Appendix 3.DOCX]

A4 Funnel Plot psychotherapy vs pharmacotherapy


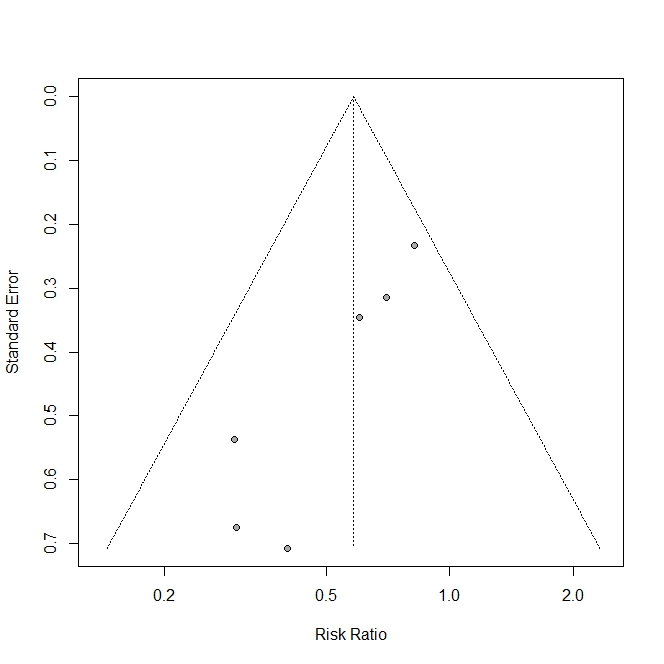

Supplement: Supplementary file 1 [file DataSheet1.zip › Appendix 4.DOCX]
